# Vaccination with an Attenuated Mutant of *Ehrlichia chaffeensis* Induces Pathogen-Specific CD4^+^ T Cell Immunity and Protection from Tick-Transmitted Wild-Type Challenge in the Canine Host

**DOI:** 10.1371/journal.pone.0148229

**Published:** 2016-02-03

**Authors:** Jodi L. McGill, Arathy D. S. Nair, Chuanmin Cheng, Rachel A. Rusk, Deborah C. Jaworski, Roman R. Ganta

**Affiliations:** 1 Center of Excellence for Vector-Borne Diseases, Department of Diagnostic Medicine/Pathobiology, College of Veterinary Medicine, Kansas State University, Manhattan, Kansas, United States of America; 2 Pathobiology Graduate Program, Department of Diagnostic Medicine/Pathobiology, College of Veterinary Medicine, Kansas State University, Manhattan, Kansas, United States of America; Upstate Medical University, UNITED STATES

## Abstract

*Ehrlichia chaffeensis* is a tick-borne rickettsial pathogen and the causative agent of human monocytic ehrlichiosis. Transmitted by the *Amblyomma americanum* tick, *E*. *chaffeensis* also causes disease in several other vertebrate species including white-tailed deer and dogs. We have recently described the generation of an attenuated mutant strain of *E*. *chaffeensis*, with a mutation in the Ech_0660 gene, which is able to confer protection from secondary, intravenous-administered, wild-type *E*. *chaffeensis* infection in dogs. Here, we extend our previous results, demonstrating that vaccination with the Ech_0660 mutant protects dogs from physiologic, tick-transmitted, secondary challenge with wild-type *E*. *chaffeensis*; and describing, for the first time, the cellular and humoral immune responses induced by Ech_0660 mutant vaccination and wild-type *E*. *chaffeensis* infection in the canine host. Both vaccination and infection induced a rise in *E*. *chaffeensis*-specific antibody titers and a significant Th1 response in peripheral blood as measured by *E*. *chaffeensis* antigen-dependent CD4^+^ T cell proliferation and IFNγ production. Further, we describe for the first time significant IL-17 production by peripheral blood leukocytes from both Ech_0660 mutant vaccinated animals and control animals infected with wild-type *E*. *chaffeensis*, suggesting a previously unrecognized role for IL-17 and Th17 cells in the immune response to rickettsial pathogens. Our results are a critical first step towards defining the role of the immune system in vaccine-induced protection from *E*. *chaffeensis* infection in an incidental host; and confirm the potential of the attenuated mutant clone, Ech_0660, to be used as a vaccine candidate for protection against tick-transmitted *E*. *chaffeensis* infection.

## Introduction

*Ehrlichia chaffeensis* is the causative agent of human monocytic ehrlichiosis (HME) [1–3]. It is an obligately intracellular Gram-negative rickettsial bacterium that is transmitted by the lone star tick, *Amblyomma americanum* [2]. White-tailed deer are the reservoir hosts for *E*. *chaffeensis*, but humans, dogs and other vertebrate species are common incidental hosts [2]. HME in people causes a life-threatening febrile illness and is associated with significant morbidity. About 40–60% of cases of HME require hospitalization, and fatality rates are estimated to be around 3% [4, 5]. There is currently no approved vaccine for use against *E*. *chaffeensis* infection in humans or animals.

Vaccine development and a detailed knowledge of immunity to *E*. *chaffeensis* infection have been limited due to lack of a robust experimental animal model for HME. Rodents are not a natural host for *E*. *chaffeensis*, and the pathogen is poorly infectious in mice, causing only transient infection in immunocompetent strains [1, 6–9]. However, through the use of immunodeficient animals, we and others have shown that clearance and protection from *E*. *chaffeensis* infection in mice relies primarily on antigen-specific CD4^+^ T cells [6, 7, 9, 10]. Infection of mice with *E*. *muris* or *Ixodes ovatus Ehrlichia* (IOE), strains closely related to *E*. *chaffeensis*, results in a systemic infection and has been used as a surrogate model of the human HME [8, 11–18]. Similar to our results with *E*. *chaffeensis* infection, clearance of a primary *E*. *muris* or IOE infection is associated with a strong, cellular immune response and production of IFNγ [8, 11, 17, 18]. Importantly, humoral immunity has also been shown sufficient in protecting mice from *Ehrlichia* infection [12, 14, 19].

Given the limitations of the mouse models of disease, our laboratory has recently turned to the use of the canine as a model for studying infection and immunity to *E*. *chaffeensis*. Dogs, like humans, are an incidental host for *E*. *chaffeensis* and are naturally infested by its tick vector, *A*. *americanum* [20]. We have recently demonstrated that *E*. *chaffeensis* infection in dogs shares similarities with infection in humans and deer, including pathogen persistence, making the canine an ideal and highly relevant model for studying host immunity [21, 22].

We recently described an approach for *E*. *chaffeensis* mutagenesis and the development of attenuated mutant strains whose growth are significantly inhibited *in vivo* in the vertebrate host [23]. Primary infection with one of these attenuated mutants, the Ech_0660 clone, promotes the development of protective immunity against a secondary challenge with virulent *in vitro* cultured *E*. *chaffeensis* in both the natural host (white-tailed deer) and an incidental host (dog) [21], suggesting our attenuated mutants are ideal candidates for vaccine development against *E*. *chaffeensis*. In this study, we tested the efficacy of vaccination with the Ech_0660 mutant against a physiologic, tick-transmitted challenge with wild-type *E*. *chaffeensis* in dogs, and for the first time conducted a detailed analysis of the humoral and cellular immune responses induced by *E*. *chaffeensis* vaccination and infection. We demonstrate that Ech_0660 mutant vaccination induces pathogen-specific antibody responses, robust CD4^+^ T cell immunity, and is efficacious against a tick-transmitted, secondary challenge with wild-type *E*. *chaffeensis*.

## Materials and Methods

### In vitro culture of *E*. *chaffeensis*

*E*. *chaffeensis* Arkansas strain (wild-type and mutant strains) and *E*. *canis* Oklahoma strain were continuously cultivated in the canine, macrophage-like DH82 cell line as described [24].

### Animals and *E*. *chaffeensis* infections

Twelve female, purebred beagle dogs of 5–6 months of age were purchased from Covance Research Products (Denver, PA). Animals were housed in a climate-controlled, biosafety level-2 facility at Kansas State University. Experimental procedures were performed in strict compliance with federal and institutional guidelines and were approved by the Kansas State University Institutional Animal Care and Use Committee.

Intravenous vaccination with attenuated *E*. *chaffeensis* transposon mutant Ech_0660 in dogs was performed as previously described [21]. Animals (n = 7) were inoculated i.v. with 2x10^8^ *E*. *chaffeensis* mutant strain, Ech_0660, organisms in 1 mL phosphate buffered saline (PBS). *Ehrlichia* organisms for vaccinations and challenge studies (below) were quantified by Taqman-based real-time PCR as we have described previously [25, 26].

Challenge infections were performed 31 days after Ech_0660 vaccination. Animals were either challenged by tick-transmission with wild-type *E*. *chaffeensis* (n = 3, group 2), by intravenous inoculation with ~2x10^8^ wild-type *E*. *chaffeensis* grown in DH82 cells (n = 2, group 1), or by intravenous inoculation with ~2x10^8^ wild-type *E*. *canis* grown in DH82 cells (n = 2, group 4). Animals that had not previously received Ech_0660 served as controls for virulent *E*. *chaffeensis* infection (n = 4, group 3). These animals were challenged via tick transmission with either wild-type *E*. *chaffeensis* or with non-attenuated Ech_0480, an isogenic mutant. We have previously demonstrated that the Ech_0480 mutant behaves similarly in culture and persists *in vivo* similar to the wild-type strain [27]; therefore, results from these animals were combined for antibody and T cell analyses (group 3). One unvaccinated dog was used as a wild-type *E*. *canis* infection control and was challenged via intravenous inoculation with ~2x10^8^ *E*. *canis* grown in DH82 cells.

Animals were humanely euthanized by barbiturate overdose and necropsies performed on day 39 post challenge. Tissues were collected for histopathology and detection of *E*. *chaffeensis* in the organs.

### Tick transmission

*E*. *chaffeensis* infected, *A*. *americanum* adult ticks were used for the tick-transmitted challenge. The tick infection was conducted as described in [27]. Briefly, nymphal ticks were needle-inoculated with 5 μl of concentrated bacterial culture containing ~5,000 wild-type *E*. *chaffeensis* or virulent Ech_0480 mutant. Nymphs were allowed to molt into adults at room temperature in a humidified chamber with 14 h daylight and 10 h darkness cycles [27]. The infection status of the needle-inoculated ticks was verified by nested PCR targeting to the Ech_1136 gene encoding for the p28-Omp 14 protein as previously described [27]. A small area on the back of the dog was shorn and a tick containment cell was affixed. Twenty-five pairs of adult ticks per dog were placed in the tick containment cell and permitted to feed for 6–7 days before removal.

### Detection of *E*. *chaffeensis* by culture recovery and molecular methods

*E*. *chaffeensis* infection was assessed in peripheral blood using culture, semi-nested PCR, and quantitative PCR as previously described [21]. At necropsy, *E*. *chaffeensis* infection was assessed in the spleen and liver using semi-nested PCR targeting to the Ech_1136 gene for *E*. *chaffeensis* or 16s rRNA gene for *E canis* as previously described [22, 27].

### Enzyme-linked immunosorbent assay (ELISA) for Total Ig and *E*. *chaffeensis*-specific IgG

Plasma samples collected prior to, and following infection were assessed by ELISA for the presence of *E*. *chaffeensis*–specific IgG as previously reported [22].

### Preparation and culture of Peripheral blood mononuclear cells (PBMC)

PBMCs were isolated by density centrifugation from buffy coat fractions of peripheral blood collected into 2x acid citrate dextrose. Cells were washed and resuspended in complete RPMI composed of RPMI-1640 (Gibco, Carlsbad, CA) supplemented with 2 mM L-glutamine, 25 mM HEPES buffer, 1% antibiotic—antimycotic solution, 50 mg/mL gentamicin sulfate, 1% nonessential amino acids, 2% essential amino acids, 1% sodium pyruvate, 50 μM 2-mercaptoethanol, and 10% (v/v) fetal bovine serum. For lymphocyte proliferation assays, cells were labeled with 1 μM CellTrace Violet (Life Technologies Inc.) per manufacturer’s instructions. Cells were cultured for 5 days at 37°C with 4x10^5^ cells /well in 96-well plates and were stimulated with 10 μg/mL host cell-free *E*. *chaffeensis* whole-cell lysate that was grown in ISE6 tick cells. As a positive control, cells were stimulated with 5 μg/mL Concanavalin A (Sigma-Aldrich). For proliferation and intracellular cytokine staining data, background (mock) responses were subtracted from the response to antigen and results are presented as change over mock.

### Antibodies and Flow Cytometry

The following monoclonal antibodies were used in these studies: mouse anti-canine CD3-FITC (clone CA17.2112), CD4-RPE or APC (clone YKIX302.9), CD8 RPE or APC (YCATE55.9), and mouse-anti-bovine IFNγ-RPE (clone CC302) all from AbD Serotec (Raleigh, NC). The bovine IFNγ-specific clone CC302 has been previously demonstrated to cross-react with canine IFNγ [28].

For surface staining, cells were resuspended at 10^7^ cells/mL in FACS buffer (0.1% NaN_3_, 10% fetal calf serum, PBS) and incubated for 20 minutes at 4°C with 10 μg/mL primary antibodies or as recommended by the manufacturer. Cells were washed and fixed in BD FACS Lysis buffer (BD Biosciences).

Intracellular cytokine staining for IFNγ was carried out using the BD Fixation and Permeabilization Solution kit (BD Biosciences). Cells were cultured with antigen for 5 days, and then Brefeldin A was added for the last 5–6 hours of incubation. Cells were surface stained and then fixed, permeabilized and stained for intracellular IFNγ (Clone CC302, 10 μg/mL) per manufacturer’s instructions.

Flow cytometry data were collected on a BD LSR Fortessa X-20 flow cytometer and analyzed using FlowJo software (Tree Star Inc., San Carlos, CA).

### ELISA for canine cytokines

PBMC culture supernatants were collected after 5 days of stimulation with 10 μg/mL host-cell free *E*. *chaffeensis* lysate. IL-4, IFNγ, and IL-17A protein concentrations were determined by commercial ELISA kit (R&D Systems, Minneapolis, MN) per manufacturer’s instructions.

### Statistics

Statistical analysis was performed using Prism v6.0f software (Graphpad Software, Inc.). To maximize power to detect differences, T cell and antibody responses were compared using an analysis of variance accounting for the repeated measures on animals over time, and the nesting of animals within each infection group as previously described [21–23]. ELISA results on cell culture supernatants from day 7-post infection were analyzed using a 1-way ANOVA with Bonferri post-test analysis.

## Results

### Attenuated mutant Ech_0660 confers protection against tick-transmitted challenge in dogs

We have demonstrated that primary infection with the attenuated Ech_0660 mutant induces protection from secondary intravenous-administered challenge with wild-type *E*. *chaffeensis* [21, 23]. To determine if the Ech_0660 mutant is also protective in a more physiologic setting of tick-transmitted challenge, we vaccinated dogs with the mutant and then performed secondary challenges on day 31-post infection. Four control dogs remained unvaccinated. Seven dogs were vaccinated i.v. with the Ech_0660 mutant organisms. Animals were monitored for the presence of *Ehrlichia* in the blood following Ech_0660 vaccination by PCR and culture recovery methods (Table 1). We have shown previously that the Ech_0660 mutant is highly attenuated and rapidly cleared from the canine host [23, 29]. In agreement with our prior studies, the Ech_0660 mutant was detected in only three animals on day 3 post vaccination. Thirty-one days after vaccination, dogs were divided into groups. Two Ech_0660 vaccinated dogs were challenged with wild-type *E*. *chaffeensis* via needle inoculation (group 1). Three vaccinated dogs were challenged with wild-type *E*. *chaffeensis* by tick transmission (group 2). The four unvaccinated control dogs were challenged via tick transmission with wild-type *E*. *chaffeensis* (n = 2) or a wild-type like, isogenic mutant strain Ech_0480 (n = 2) (group 3). We have previously demonstrated that the Ech_0480 mutant behaves like the wild-type strain of *E*. *chaffeensis*, displaying similar persistence in the vertebrate host (23); therefore we have combined the data for these two control groups (group 3).

**Table 1:** Infection status of dogs vaccinated with attenuated mutant Ech_0660.

	Days Post Vaccination							
	0	3	8	11	14	21	28	31
Ech_0660_1^a^	-	-	-	-	-	-	-	-
Ech_0660_2	-	-	-	-	-	-	-	-
Ech_0660_3	-	c^b^	-	-	-	-	-	-
Ech_0660_4	-	-	-	-	-	-	-	-
Ech_0660_5	-	-	-	-	-	-	-	-
Ech_0660_6	-	c	-	-	-	-	-	-
Ech_0660_7	-	c	-	-	-	-	-	-

^a^ Seven dogs were inoculated i.v. with 2x10^8^ *E*. *chaffeensis* mutant Ech_0660 organisms.

^b^ Dogs were tested at the indicated time points for *E*. *chaffeensis* organisms in the blood by PCR (p) and culture recovery methods (c) as described [21].

*E*. *chaffeensis* infection in dogs varies from subclinical infection to severe systemic disease. Mild clinical signs may manifest as low-grade fever or thrombocytopenia, as we and others have previously reported [21–23, 30]. In this experiment, we did not observe significant clinical disease in vaccinated or control dogs (data not shown). *E*. *chaffeensis* infection was monitored in the blood after secondary challenge using nested PCR and culture recovery methods. The results are shown in Table 2. Dogs that were vaccinated and challenged with wild-type *E*. *chaffeensis* by needle inoculation (group 1) were protected from infection, as evidenced by testing positive for infection in the blood only twice in one animal on days 8 and 11 post challenge (12.5% of the time), and testing negative for the organism in the spleen and liver at the time of necropsy. Vaccinated dogs that were challenged via tick-transmission (group 2) were also protected from secondary challenge. This group tested positive for *Ehrlichia* in the blood 29.1% of the time (7 out of 24 total blood samples tested). However, no blood positives were obtained after day 15 post challenge. All animals were also negative for the organism in the spleen and liver at the time of necropsy. This result suggests that while dogs may develop ehrlichemia early following infection, vaccination with the Ech_0660 mutant promotes protection from long-term pathogen persistence in the blood and organs. In contrast, unvaccinated control dogs (group 3) displayed persistent infection, testing frequently positive for the organism throughout the 31 days of assessment (about 34.3% of the time: 11 out of 32 samples tested) and testing positive for the organism in the tissues at necropsy.

**Table 2:** Infection status of Ech_0660 vaccinated dogs and unvaccinated control dogs following wild-type *E*. *chaffeensis challenge*.

	**Days Post Challenge: WT *E*. *chaffeensis* by needle transmission**								**Necropsy**^e^		
**Group 1**^a^	**0**	**4**	**8**	**11**	**15**	**22**	**29**	**36**	**blood**	**spleen**	**liver**
Ech_0660_1	-	-	-	-	-	-	-	-	-	-	-
Ech_0660_2	-	-	p^d^	c	-	-	-	-	-	-	-
	**Days Post Challenge: WT *E*. *chaffeensis* by tick transmission**										
**Group 2**^b^	**0**	**4**	**8**	**11**	**15**	**22**	**29**	**36**	**blood**	**spleen**	**liver**
Ech_0660_3	-	-	c	-	c	-	-	-	-	-	-
Ech_0660_4	-	-	c	-	c	-	-	-	-	-	-
Ech_0660_5	-	-	p/c	p	c	-	-	-	-	-	-
	**Days Post Challenge: WT *E*. *chaffeensis* or Ech_0480 by tick transmission**										
**Group 3**^c^	**0**	**3**	**7**	**10**	**14**	**17**	**24**	**31**	**blood**	**spleen**	**liver**
Wild-type_1	-	-	p	-	c	-	-	p	-	-	+
Wild-type_2	-	-	p	-	c	p	-	-	-	-	-
Ech_0480_1	-	-	-	-	c	-	-	-	-	-	+
Ech_0480_2	-	p	-	-	c	-	p	p	-	+	-

^a^ Dogs from Table 1 were challenged 31 days after vaccination. Animals were challenged via i.v. inoculation with 2x10^8^ wild-type *E*. *chaffeensis* organisms

^b^ Dogs from Table 1 were challenged 31 days after vaccination. Animals were challenged via tick-transmission with wild-type *E*. *chaffeensis* organisms

^c^ Unvaccinated control dogs were challenged with 2x10^8^ wild-type *E*. *chaffeensis* organisms or 2x10^8^ Ech_0480 mutant *E*. *chaffeensis* organisms

^d^ Dogs were tested at the indicated time points for *E*. *chaffeensis* organisms in the blood by PCR (p) and culture recovery methods (c) as described [21]. Animals testing positive by both methods are indicated by (p/c)

^e^ Animals were euthanized and necropsied on day 39 post challenge.

To determine if Ech_0660 mutant inoculation protects dogs against a heterologous challenge, we challenged the remaining two Ech_0660 vaccinated animals with a closely related *Ehrlichia* organism, *E*. *canis*, by needle inoculation (group 4). One unvaccinated control animal was also infected with wild-type *E*. *canis* by needle inoculation. Dogs in group 4 tested positive for infection in the blood 81.2% of the time (13 out of 16 samples tested), similar to the unvaccinated control animal, suggesting that the Ech_0660 mutant is not protective against heterologous *E*. *canis* infection (Table 3). Importantly, as only two animals were included in this group, additional experiments will be necessary to confirm this result and to achieve statistical significance.

**Table 3:** Infection status of Ech_0660 vaccinated dogs and unvaccinated control dog following wild-type *E*. *canis* challenge.

	**Days Post Challenge: WT *E*. *chaffeensis* by needle transmission**								**Necropsy**^d^		
**Group 4**^a^	**0**	**4**	**8**	**11**	**15**	**22**	**29**	**36**	**blood**	**spleen**	**liver**
Ech_0660_6	-	p/c^c^	p/c	p/c	p/c	p/c	p/c	p/c	p/c	-	+
Ech_0660_7	-	p/c	-	p/c	p/c	p/c	p/c	p/c	p/c	-	-
	**Days Post Challenge: WT *E*. *chaffeensis* by tick transmission**										
**Control**^b^	**0**	**3**	**7**	**10**	**14**	**17**	**24**	**31**	**blood**	**spleen**	**liver**
E_canis_1	-	p/c	p/c	p/c	p/c	p/c	p/c	p/c	p/c	+	+

^a^ Dogs from Table 1 were challenged 31 days after vaccination. Animals were challenged i.v. with 2x10^8^ wild-type *E*. *canis* organisms

^b^ Unvaccinated control dog was challenged with ~2x10^8^ wild-type *E*. *canis* organisms

^c^ Dogs were tested at the indicated time points for *E*. *canis* organisms in the blood by PCR (p) and culture recovery methods (c) as described [22, 26]. Animals testing positive by both methods are indicated by (p/c)

^d^ Animals were euthanized and necropsied on day 39 post challenge

### Ech_0660 vaccination and wild-type *E*. *chaffeensis* infection induce pathogen-specific antibody production

**Fig 1:**
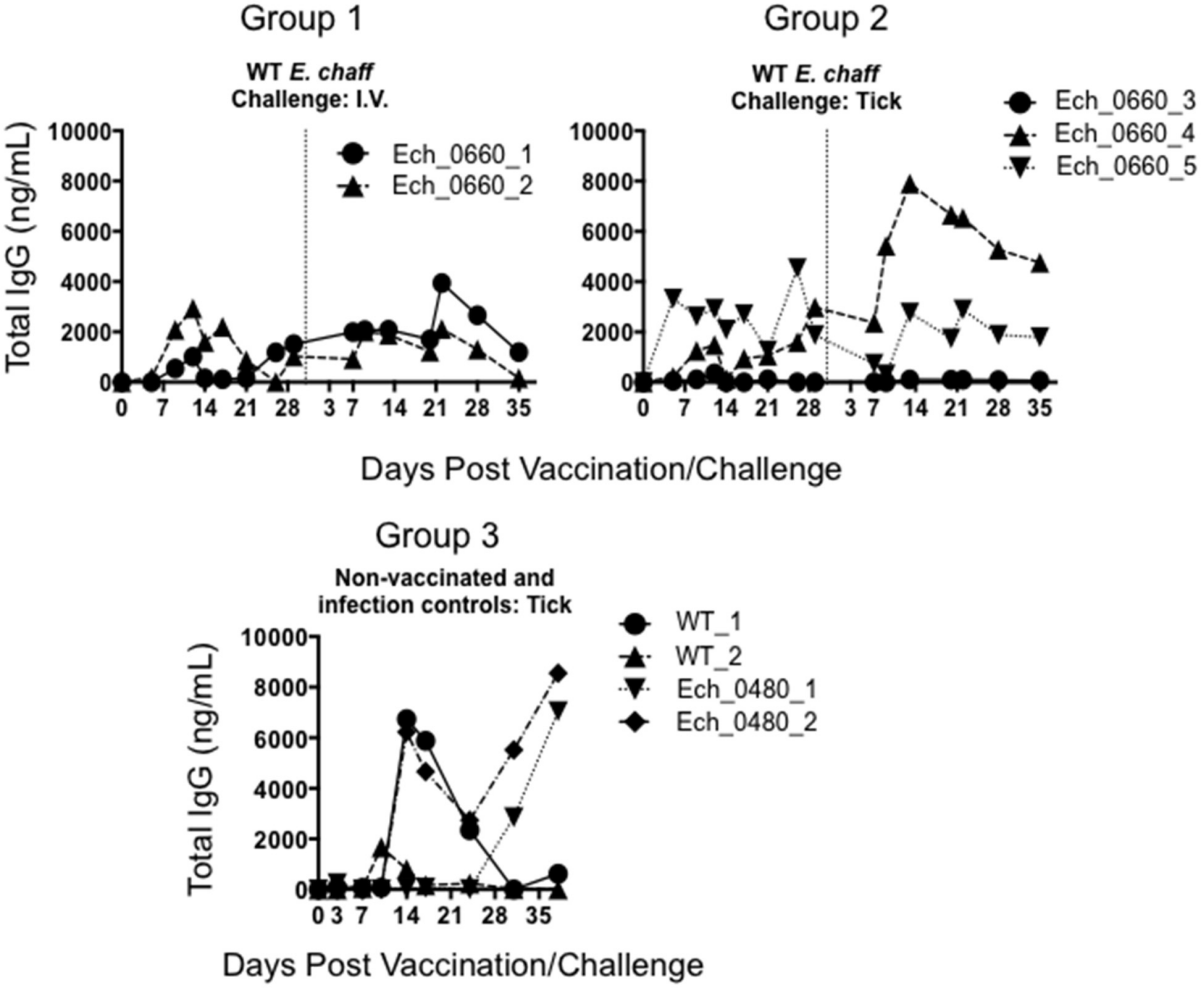
*E*. *chaffeensis*-specific IgG response following Ech_0660 vaccination and secondary challenge with wild-type *E*. *chaffeensis*. Total *E*. *chaffeensis*-specific IgG was measured in the plasma at multiple time points by ELISA in dogs vaccinated with the Ech_0660 mutant and challenged with wild-type *E*. *chaffeensis* via needle inoculation (group 1), or vaccinated with Ech_0660 and challenged with wild-type *E*. *chaffeensis* via tick-transmission (group 2). Unvaccinated control dogs were infected with wild-type *E*. *chaffeensis* or the non-attenuated Ech_0480 mutant via tick-transmission (group 3). Each line is representative of a single animal.

Plasma samples from vaccinated and unvaccinated control dogs were evaluated by ELISA for total *E*. *chaffeensis*-specific IgG. Vaccination resulted in an increase in *E*. *chaffeensis* specific IgG in 4 out of 5 dogs (Fig 1). We also observed an increase in pathogen-specific IgG following wild-type challenge in both vaccinated dogs and unvaccinated controls. There were no significant differences in the humoral response between vaccinated and control dogs after secondary challenge.

### Vaccination and wild-type *E*. *chaffeensis* challenge induces antigen-dependent CD4^+^ T cell responses

**Fig 2:**
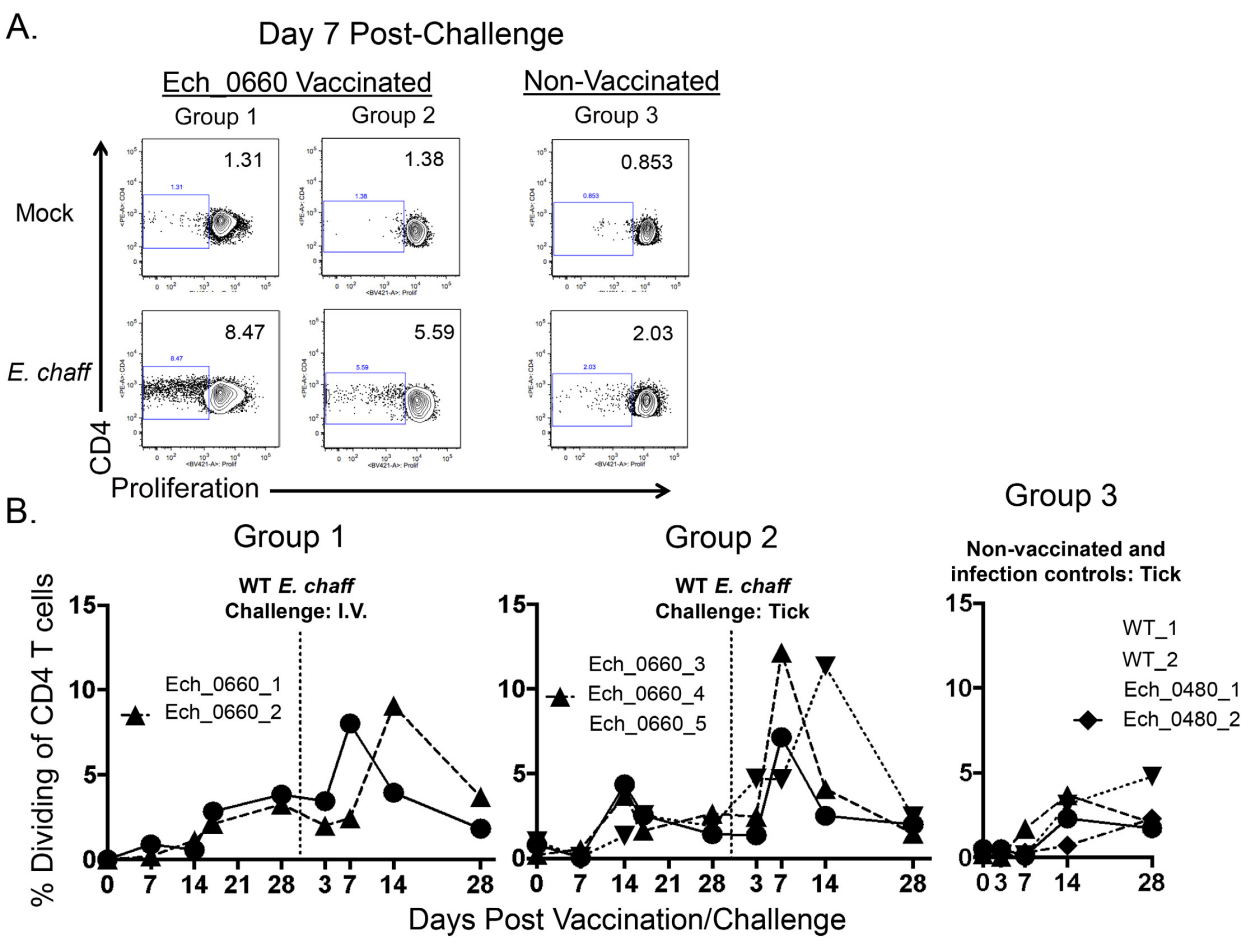
CD4^+^ T cells from Ech_0660 mutant vaccinated and wild-type *E*. *chaffeensis* infected animals proliferate in response to *E*. *chaffeensis* antigen. PBMC from dogs vaccinated with Ech_0660 and challenged with wild-type *E*. *chaffeensis* via needle inoculation (group 1, left panels), vaccinated with Ech_0660 and challenged with wild-type *E*. *chaffeensis* via tick inoculation (group 2, middle panels), or unvaccinated and infected with wild-type *E*. *chaffeensis* or Ech_0480 via tick inoculation (group 3, right panels) were labeled with Cell Trace Violet, then cultured for 5 days at 4x10^6^ cells/mL in the presence or absence of 10 ug/mL *E*. *chaffeensis* host-cell free lysate grown in the tick ISE6 cell line. On day 5, CD4^+^ T cells were analyzed by flow cytometry for Cell Trace Violet dilution as a measure of proliferation. (A) Representative Cell Trace Violet dilution profiles, gated on total live cells and total CD3^+^CD4^+^ T cells. (B) The percentage of CD4^+^ T cells that have proliferated in response to *E*. *chaffeensis* antigens as measured over the course of the experiment. The background (mock stimulated) proliferation was subtracted, and results represent change over mock. Each line is representative of a single animal.

We next measured *E*. *chaffeensis*-specific CD4^+^ T cell recall responses in peripheral blood from vaccinated and control dogs. PBMC were labeled with Cell Trace Violet, stimulated with host cell-free *E*. *chaffeensis* whole cell lysate, and then analyzed by flow cytometry. Antigen-dependent CD4^+^ T cells were identified based upon proliferation in response to *E*. *chaffeensis* antigen as determined by dilution of the Cell Trace Violet dye. Fig 2A shows representative dilution profiles of mock and antigen-stimulated CD4^+^ T cells from one animal per group on day 7 post secondary challenge. The numbers depicted in Fig 2A represent the percent of proliferating CD3^+^CD4^+^ cells contained within each gate. Fig 2B shows the percentage of CD4^+^ T cells dividing in response to antigen that was measured in all animals over the course of the experiment. Background levels of proliferation were subtracted from these values, and results represent change in proliferation over mock stimulated cultures. We observed an increase in the percentage of CD4^+^ T cells that divided in response to *E*. *chaffeensis* antigen in PBMC collected on day 14–17 post inoculation with the Ech_0660 mutant. This percentage was further increased following wild-type *E*. *chaffeensis* challenge. Vaccinated animals displayed significantly higher percentages of proliferating *E*. *chaffeensis* antigen-dependent CD4^+^ T cells compared to unvaccinated dogs (Fig 2B, p = 0.0081).

**Fig 3:**
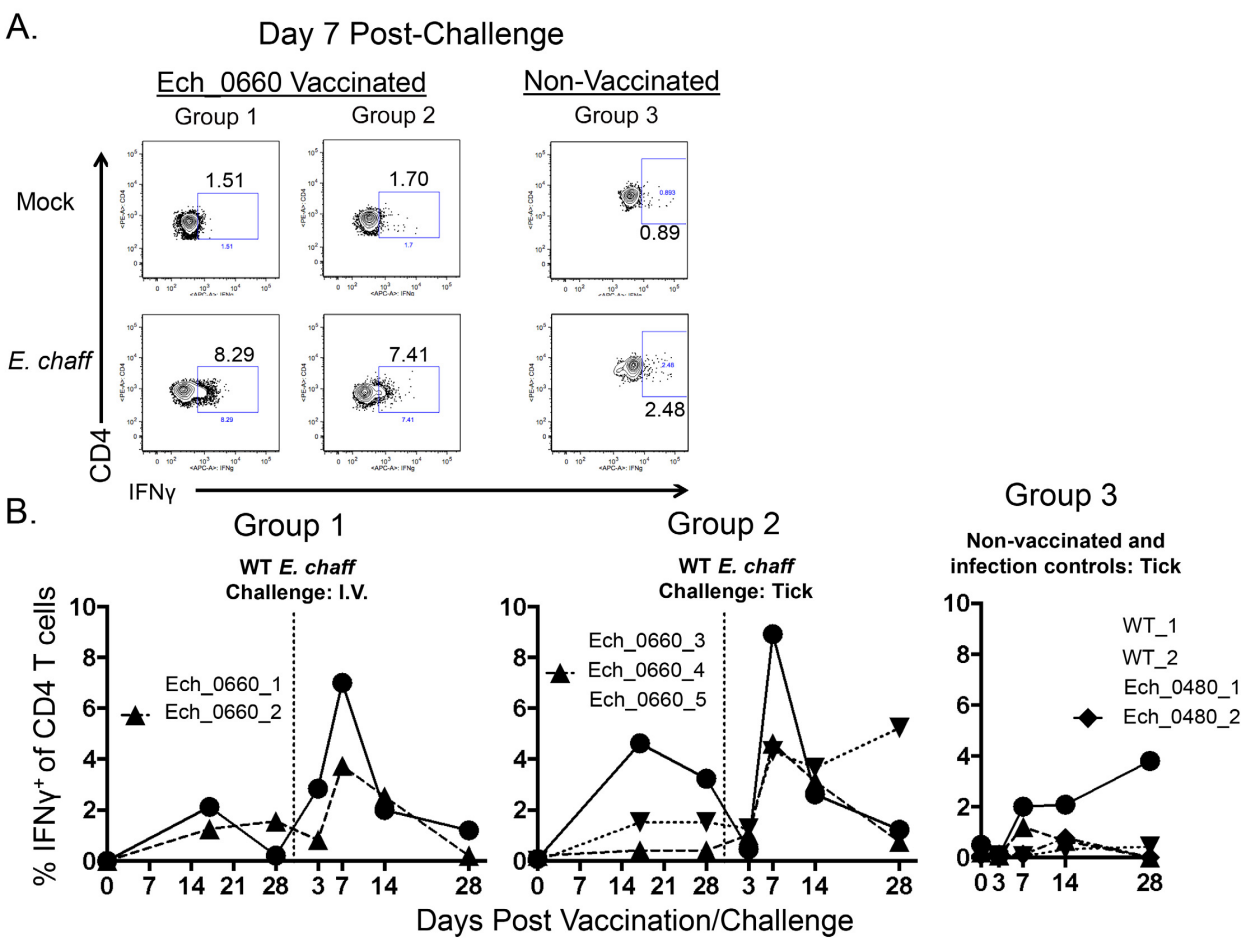
CD4^+^ T cells from Ech_0660 mutant vaccinated and wild-type *E*. *chaffeensis* infected animals produce IFNγ in response to *E*. *chaffeensis* antigen. PBMC from dogs vaccinated with the Ech_0660 mutant and challenged with wild-type *E*. *chaffeensis* (groups 1–3, as in Fig 2) were cultured for 5 days at 4x10^6^ cells/mL in the presence or absence of 10 ug/mL *E*. *chaffeensis* host-cell free lysate grown in the tick ISE6 cell line. On day 5, brefeldin A was added for the last 6 hours of culture. CD4^+^ T cells were stained for intracellular expression of IFNγ and analyzed by flow cytometry. (A) Representative flow plots from animals in groups 1, 2 and 3, gated on total live cells and total CD3^+^CD4^+^ T cells. (B) The percentage of IFNγ^+^ cells of total CD4^+^ T cells in the blood measured over the course of the experiment. Background (mock stimulated) IFNγ production was subtracted, and results represent change over mock.

We also measured antigen-dependent IFNγ production by CD4^+^ T cells in the blood using intracellular cytokine staining. Fig 3A shows representative flow plots of mock and antigen-stimulated CD4^+^ T cells gated on IFNγ^+^ cells. Flow plots are from one animal per group on day 7 post secondary challenge. Fig 3B shows the combined results from all animals. We observed significantly increased percentages of CD4^+^ T cells producing IFNγ in response to *E*. *chaffeensis* antigen in samples from vaccinated animals, compared to unvaccinated controls (Fig 3B, p = 0.0025).

**Fig 4:**
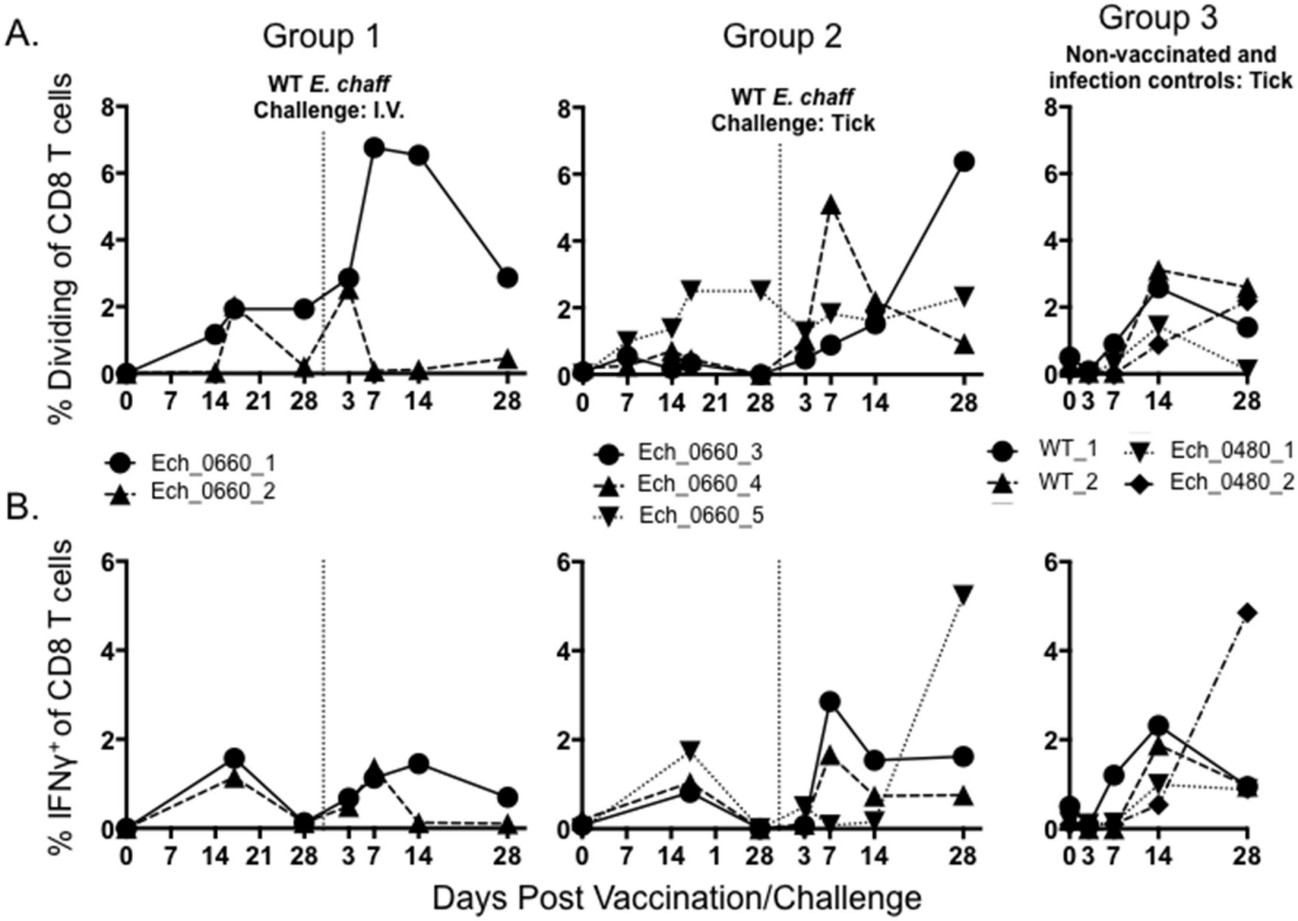
CD8^+^ T cells from Ech_0660 vaccinated and wild-type *E*. *chaffeensis* infected animals proliferate and produce IFNγ in response to *E*. *chaffeensis* antigen. CD8^+^ T cell proliferation and IFNγ production were measured using similar approaches as in Figs 2 and 3. PBMC from dogs in groups 1–3 were cultured for 5 days at 4x10^6^ cells/mL in the presence or absence of 10 ug/mL *E*. *chaffeensis* host-cell free lysate. On day 5 of culture, CD8^+^ T cells were analyzed by flow cytometry for (A) proliferation as measured by Cell Trace Violet dilution; and (B) intracellular production of IFNγ. The frequencies of responding CD8^+^ T cells were measured over the course of the experiment. Results were gated on total live cells and total CD3^+^CD8^+^ T cells. Background (mock stimulated) proliferation or IFNγ production was subtracted and results represent change over mock.

*E*. *chaffeensis*-specific CD8^+^ T cell proliferation and IFNγ production was also measured by flow cytometry (Fig 4). Neither vaccination nor infection with wild-type *E*. *chaffeensis* induced a significant CD8^+^ T cell response as measured by proliferation assay (Fig 4A) or by intracellular cytokine staining for IFNγ (Fig 4B). While we observed a trend towards an increase in the CD8^+^ T cell response from vaccinated dogs following secondary challenge, this response was not significant over that observed in control dogs (group 3).

**Fig 5:**
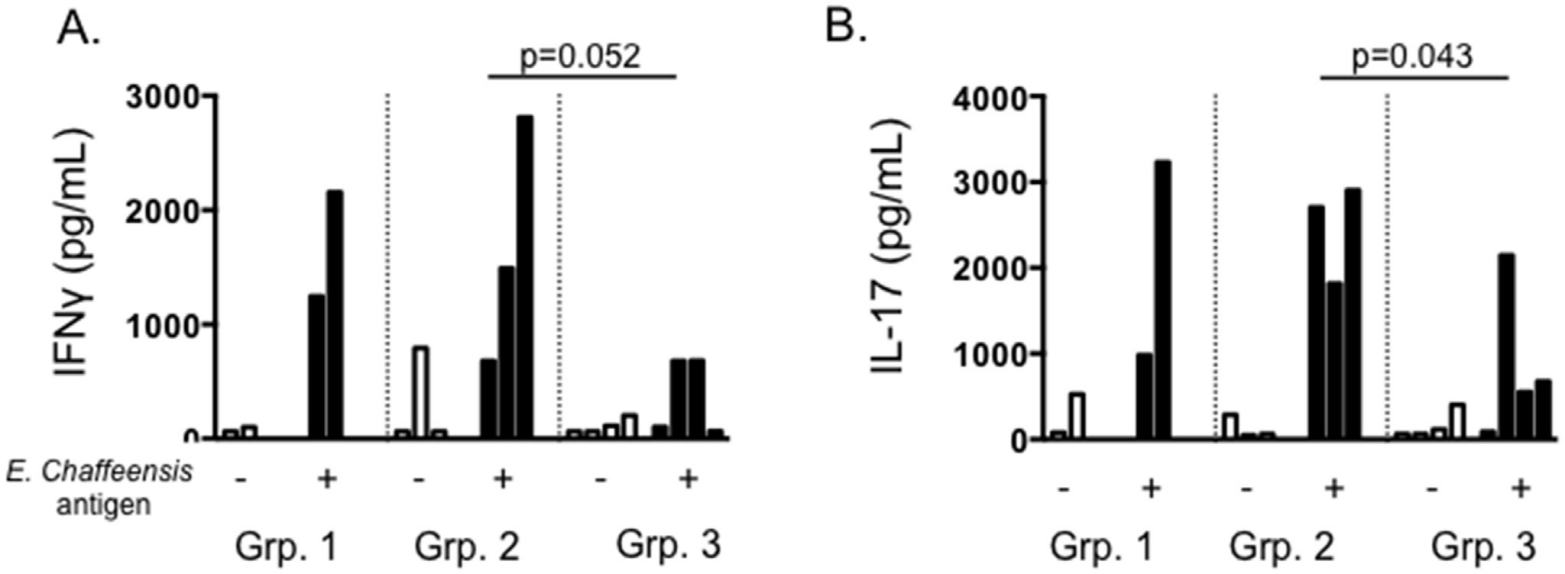
PBMC from Ech_0660 vaccinated and wild-type *E*. *chaffeensis* infected animals secrete IFNγ and IL-17 in response to *E*. *chaffeensis* antigen. PBMC from dogs vaccinated with Ech_0660 and challenged with wild-type *E*. *chaffeensis* (groups 1–3, as in Fig 2) were collected on day 7 post-secondary challenge with wild-type *E*. *chaffeensis*. PBMC were cultured for 5 days at 4x10^6^ cells/mL in the presence or absence of 10 ug/mL *E*. *chaffeensis* host-cell free lysate grown in the tick ISE6 cell line. On day 5, cell culture supernatants were collected and later analyzed by ELISA for secretion of (A) IFNγ, (B) IL-17, and IL-4 (not shown). Each bar is representative of a single animal.

ELISAs were used to measure Th1, Th2 and Th17 cytokines secreted by PBMC in recall responses to *E*. *chaffeensis* antigen. PBMC from Ech_0660 vaccinated animals secreted IFNγ (Th1) in response to *E*. *chaffeensis* antigen (Fig 5A); and this response was significantly increased over the response from unvaccinated control dogs. We did not observe appreciable IL-4 (Th2) production by PBMC from vaccinated or control dogs; however, all three groups mounted a vigorous IL-17 response to *E*. *chaffeensis* antigen (Fig 5B). IL-17 production by cells from Ech_0660 vaccinated dogs was significantly increased over unvaccinated controls.

## Discussion

We recently reported the ability of a clonally purified transposon mutant of *E*. *chaffeensis*, Ech_0660, to induce protection from secondary, intravenous challenge with wild-type *E*. *chaffeensis* in the incidental canine host [21]. In the current study, we demonstrate that Ech_0660 mutant vaccinated animals are also protected from a physiologically relevant, tick-transmitted infection challenge. In this study, we also report the induction of robust *E*. *chaffeensis*-specific antibody and CD4^+^ T cell responses in animals receiving Ech_0660 mutant vaccination and secondary challenge.

In nature, *E*. *chaffeensis* is transmitted to a host when an infected *A*. *americanum* tick takes a blood meal [2]. Previous studies have shown that the route of inoculation of vector-borne pathogens such as *Ehrlichia* can have a profound effect on vaccine-induced protection and ultimately disease outcome. For example, a recent study by Pretorius *et al*. showed that an experimental prime/boost DNA vaccine regimen afforded 100% protection to sheep challenged via needle-transmitted *Ehrlichia ruminantium* challenge; while the same regimen provided less than 20% protection from a physiologic tick-transmitted *E*. *ruminantium* challenge [31]. While a number of factors were at play in this study, the authors attribute the vaccine failure to fundamental differences between tick-transmitted and needle-transmitted challenge infections. In our study, we analyzed the immune response to dogs challenged with wild-type *E*. *chaffeensis* via needle-inoculation or via a more physiologic route of tick-transmitted challenge. We did not observe obvious differences in the immunologic parameters we measured in this study between the two routes of inoculation, including the magnitude of the humoral or cell-mediated immune response. However, we used only 2 animals in the group receiving *E*. *chaffeensis* via needle-inoculation (group 2), because this route of inoculation was not the primary focus of this study. Further, we previously reported that Ech_0660 vaccination promoted protection against intravenous inoculation challenge in our recent report [21]. It is possible that critical differences may exist between the immune response induced by intravenous infection compared to tick-transmitted challenge. We have previously demonstrated in studies using the murine model of *E*. *chaffeensis* infection that the route of inoculation, and the source of inoculum (e.g. organisms grown in tick cells vs. those grown in canine macrophage cells) can have a significant effect on the specificity and nature of the immune response [25]. We hypothesize this effect is due to the differential expression of *Ehrlichia* outer membrane proteins expressed during growth in tick cells vs. macrophage cells. In support of this theory, we have demonstrated that approximately 50% of *E*. *chaffeensis* proteins are expressed in a host-cell specific manner [32]. In addition, ticks employ a number of well-described immunomodulatory factors that play a critical role in allowing vector-borne pathogens to establish infection (reviewed in [33]). Amongst these strategies: inhibition of host inflammatory cytokine production such as IL-1, IL-12, TNF-α and IFNγ inhibition of lymphocyte proliferative responses; and downregulation of macrophage nitric oxide production.

We demonstrate here that *E*. *chaffeensis* vaccination and challenge in dogs induces robust antigen-dependent CD4^+^ T cell responses, but not a significant antigen-dependent CD8^+^ T cell response. This result may be due to large animal-to-animal variability, or it may be that CD8^+^ T cell responses are not a major component of the *Ehrlichia*-specific immune response in dogs. In support of our observations, studies from mice agree that CD4^+^ T cell immunity is critical for the response to *Ehrlichia*, while the role of CD8^+^ T cells is less clear [6, 11, 13, 14, 17]. We observe only minor CD8^+^ T cell responses following primary *E*. *chaffeensis* infection in C57BL/6 mice [6], and a similar result is observed during IOE infection of C57BL/6 mice [11]. In contrast, cytotoxic T cells appear critical during *E*. *muris* infection of C3H/HeN mice, as infection of animals lacking MHC class I results in over 80% lethality [14]. In the future, it will be important to more clearly define the contribution of CD8^+^ T cells in the response to *E*. *chaffeensis* vaccination and protection in the natural host.

*E*. *canis* is genetically related to *E*. *chaffeensis*, and the primary etiologic agent of canine monocytic ehrlichiosis [34]. In this study, we tested the ability of our *E*. *chaffeensis* mutant vaccine strain to promote protection from heterologous *E*. *canis* challenge. We did not observe protection from *E*. *canis*, as two dogs that received the Ech_0660 mutant tested positive for ehrlichemia for the duration of the study. While this result is disappointing, similar examples of poor protection from heterologous challenge have been previously reported for the rickettsials, including lack of protection between strains of *E*. *ruminantum* [35], of *Anaplasma phagocytophilum* [36, 37] and of *E*. *canis* [38, 39].

To the best of our knowledge, ours is the first report of IL-17 production in the context of *Ehrlichia* infection. IL-17 is a pro-inflammatory cytokine that is primarily produced by activated CD4^+^ and γδ T cells [40]. It induces expression of a number of chemotactic factors, particularly IL-8, and is critical for the recruitment and activation of neutrophils. A balanced IL-17 response seems favorable for control of a number of intracellular bacterial infections, but excessive IL-17 contributes to damaging immunopathology. For example, in a mouse model of *Mycobacterium bovis* infection, IL-17 is essential for pathogen control and for appropriate maturation of granulomas [41]; while excessive IL-17 promotes exacerbated inflammation and increased mortality [42]. Similarly, in a mouse model of *Chlamydia muridarum* infection, IL-17 contributes to disease pathology, but is also essential for protection from secondary infection [43, 44]. Our results suggest that IL-17 production may correlate with protection from wild-type *E*. *chaffeensis* infection in a canine host. However, as observed during other intracellular infections, it is probable that balance is critical for host defense, and excessive IL-17 production may be detrimental to the host. In support of this, a recent report in humans showed that elevated levels of IL-8 are associated with fatal HME [45]. While this study did not examine expression of IL-17, it is a mechanism that should be considered in light of our recent findings. Future studies will be required to determine the role of IL-17 and Th17 immunity in protection or possibly immunopathology during *E*. *chaffeensis* infection in a natural host.

In conclusion, we demonstrate that vaccination with the live, attenuated mutant Ech_0660 induces pathogen-specific humoral and cellular immunity, and protection from tick-transmitted *E*. *chaffeensis* infection in a physiologic host. This report represents the first detailed analysis of the immune responses induced by vaccination and infection in a natural host for *E*. *chaffeensis*, and a critical first step in developing our understanding of immunity to this important, emerging pathogen.
